# Different metabolic pathways associated with total cortisol exposure and the cortisol time profile: a randomized crossover trial

**DOI:** 10.1038/s41598-026-37816-0

**Published:** 2026-02-12

**Authors:** Johanna McQueen, Terence Garner, Dimitrios Chantzichristos, Hans Lennernäs, Stéphanie Espiard, Oskar Ragnarsson, Ragnhildur Bergthorsdottir, Stanko Skrtic, Adam Stevens, Gudmundur Johannsson

**Affiliations:** 1 Department of Internal Medicine and Clinical Nutrition, Institute of Medicine at Sahlgrenska Academy, University of Gothenburg, Gothenburg, Sweden; 2 Endocrinology, Diabetology and Metabolism, Sahlgrenska University Hospital, Gothenburg, Sweden; 3 Division of Developmental Biology and Medicine, Faculty of Biology, Medicine and Health, University of Manchester, Manchester, United Kingdom; 4 Department of Pharmaceutical Biosciences, Uppsala University, Uppsala, Sweden; 5 Department of Endocrinology, Diabetology and Metabolism, Huriez Hospital, Lille University Hospital, Lille, France; 6 Wallenberg Center for Molecular and Translational Medicine, University of Gothenburg, Gothenburg, Sweden; 7 Medical & Science, Cardiovascular, Kidney and Alzheimer Disease, Novo Nordisk Development, Copenhagen, Denmark

**Keywords:** Adrenal insufficiency, Glucocorticoid replacement therapy, Total cortisol exposure, Cortisol time profile, Metabolomics, Pathway analysis, Metabolomics, Steroid hormones, Metabolism, Biomarkers

## Abstract

Excess cortisol exposure and disruption of its circadian pattern have both been linked to adverse health outcomes; however, whether distinct metabolic signatures differentiate total cortisol exposure from cortisol secretion dynamics remains unclear. This study aimed to identify metabolites and metabolic pathways uniquely associated with total cortisol exposure and/or variation of the cortisol time profile. In a randomized, 12-week, cross-over trial 18 adults with primary adrenal insufficiency (AI) received the same total daily dose of hydrocortisone (HC) administered as a once-daily (OD) dual-release tablet and conventional HC tablets 3 times daily (TID). Serum and urine samples were collected during 24h in-house standardized pharmacokinetic sampling days and metabolites were detected using liquid and gas chromatography-mass spectrometry (LC-MS and GC-MS). Total cortisol exposure was quantified as the area under the serum cortisol concentration-time curve and variability in the cortisol time profile was assessed by calculating the lag–1 autocorrelation from serum cortisol concentrations over 24 hours. Compared with OD dosing, TID administration resulted in a 20% higher total cortisol exposure and a greater variability in the cortisol time profile. In total, 2406 metabolites were detected. Pathway analysis of serum metabolites uniquely correlated with total cortisol exposure were involved in amino acid metabolism - including arginine, tryptophan and glutamate pathways - as well as glycerolipid metabolism. In contrast, metabolites uniquely associated with variability of the cortisol time profile were mapped to primary bile acid biosynthesis and cysteine-methionine metabolism. We identified distinct groups of metabolites and metabolic pathways that specifically correlate with either overall serum cortisol exposure or variability in its time profile, indicating that cortisol dose exposure and the circadian dynamics may exert independent metabolic and regulatory effects in humans, with potential implications for personalized hydrocortisone therapy.

## Introduction

Glucocorticoids (GCs) are essential for life and play an important role in a wide range of physiological processes in the human body, including intermediary metabolism, immune response, and cardiovascular and brain function^1^. Under normal physiological conditions, GC secretion occurs in a circadian rhythm, governed by the hypothalamic-pituitary-adrenal axis (HPA), with a serum cortisol peak in the early morning followed by a gradual decline toward a nadir at midnight^2^. Moreover, cortisol secretion occurs as pulsations with higher ultradian peaks in early morning followed by almost unnoticeable peaks in the evening^3^. Tissue sensitivity to GCs is also regulated in a circadian pattern, with increased cortisol sensitivity at night due to interaction between clock genes and the GC receptors^4,5^. Both insufficient and excessive total GC exposure are associated with severe clinical features and poor patient outcomes, as seen in patients with adrenal insufficiency (AI) and Cushing’s syndrome^6–9^. Independently, disruption of the circadian rhythm, whether due to altered timing or rhythmicity of cortisol exposure, has additionally been linked to impaired glucose tolerance, elevated blood pressure and increased risk of cardiovascular events^4^. Further, an abnormal cortisol exposure time profile has been shown to disrupt circadian gene expression, negatively affecting brain and immune cell function^10,11^.

Despite optimization using current knowledge and available treatment options, patients with AI on GC replacement therapy continue to experience compromised survival, increased cardiometabolic risk factors, a higher incidence of severe infections, and an impaired health-related quality of life^9,12,13^. Whether these effects stem from excessive total daily GC doses or a non-physiological cortisol time profile, or both, is still uncertain. A once-daily (OD) dual-release hydrocortisone (HC) tablet produces a markedly different serum cortisol profile in these patients, compared with conventional immediate-release tablets, which require multiple daily administrations^14^. In contrast to the pronounced peaks and troughs seen with conventional HC, caused by rapid absorption and clearance after each dose, the OD formulation provides a smoother profile, with a rapid morning rise after intake followed by a gradual decline throughout the day and reduced intra-day variability. This treatment improves the metabolic risk profile in these patients with reduction in body weight and blood pressure^15,16^. In addition, OD treatment has been shown to normalize clock gene expression as well as improve the urinary cortisol metabolome in patients with AI^11,17^. Whether these beneficial effects are due to an improved serum cortisol profile or the simultaneously observed reduction in total cortisol exposure with OD dual-release HC tablets in these studies, remains unclear.

Metabolomics is the study of metabolites, small molecular compounds that are intermediates or end-products of intra- and extracellular metabolism^18^. It is a rapidly developing field that combines advanced analytical techniques with in-depth statistical approaches to improve understanding of biological processes. Metabolomics has recently been used to study the metabolic aspects of GC replacement therapies in patients with various forms of AI^19–21^. These studies have found a GC dose-dependent effect on specific amino acids such as tryptophan and tyrosine, fatty acids, some acyl carnitines, and the pyrimidine base uracil.

A more mechanistically driven approach using metabolomic analysis may help clarify whether total cortisol exposure and/or variability in the cortisol time profile, independently modulate different metabolic pathways. This would increase the understanding of outcomes related to different GC replacement regimens and in diseases and conditions with a non-physiological cortisol time profile. The aim of this study was to identify metabolites and metabolic pathways associated with total cortisol exposure and the variability of the cortisol time profile, independently. For this purpose, we compared the serum and urinary metabolome in patients with Addison’s disease who received treatment with both conventional HC tablets three times daily (TID) and dual-release HC tablets OD, in a randomized, crossover, clinical trial.

## Results

### Patient characteristics, clinical outcomes, and serum cortisol features

Eighteen patients (10 males and 8 females) with primary AI were included in the analysis. Their mean (SD) age was 48.3 (12.0) years and their mean (SD) daily HC replacement dose was 32 (5) mg. All patients received daily fludrocortisone treatment. Six patients (33.3%) had concomitant treatment with levothyroxine, three (16.7%) had hypertension, and four (22.2%) had diabetes mellitus (Table 1).

**Table 1:** Patient demographics and baseline clinical characteristics.

Parameter	Value
Age, mean (SD), y	48.3 (12.0)
Sex	
Male	10 (55.6)
Female	8 (44.4)
Hydrocortisone replacement dose, mean (SD), mg	32 (5)
Fludrocortisone replacement	18 (100)
Levothyroxine treatment	6 (33.3)
Hypertension	3 (16.7)
Diabetes mellitus	4 (22.2)
Type 1	3 (16.7)
Type 2	1 (5.6)

Results are shown as n (%) unless otherwise specified.

Abbreviations: y, year; SD, standard deviation.

**Figure 1:**
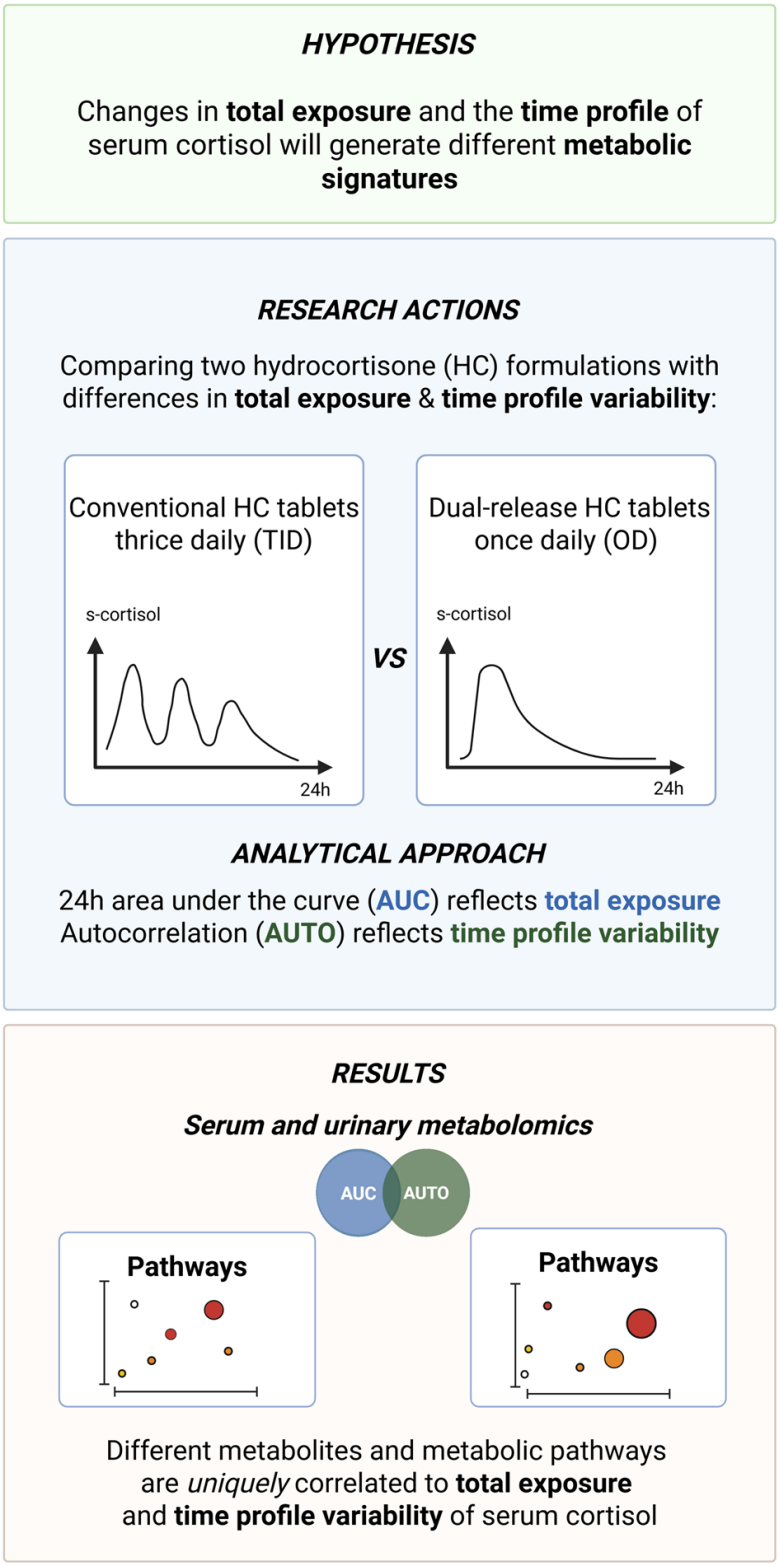
Schematic flow chart of the study approach including illustrative serum cortisol profiles based on previously published pharmacokinetic data^15^.

**Figure 2:**
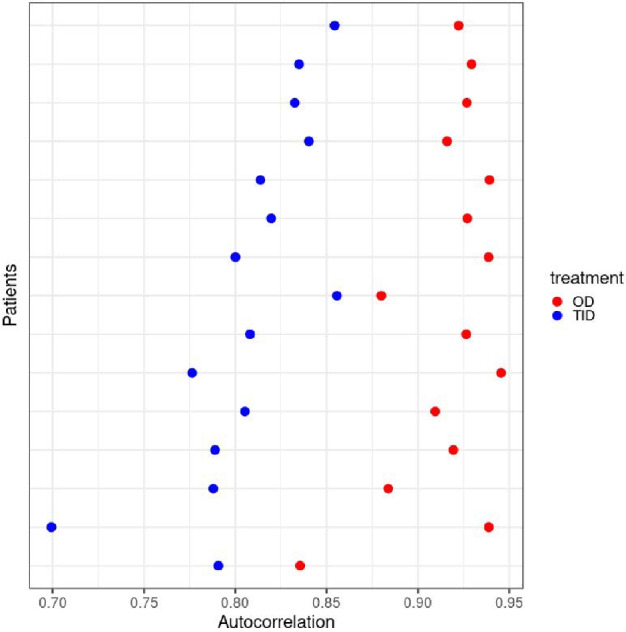
Scatterplot showing the autocorrelation values for each patient during the two different treatment periods (OD and TID). The variability of the serum cortisol profile decreases with autocorrelations closer to 1. The serum cortisol time profile variability was significantly lower during OD compared to TID (*P* = 2.2 × 10^–16^), which is reflected by higher autocorrelation values. Abbreviations: OD, once daily; TID, 3 times daily.

All patients completed TID treatment (n = 18) and 16 completed OD treatment. Mean body mass index (BMI), systolic and diastolic blood pressure and glycated hemoglobin (HbA_1c_) were reduced after 12 weeks of OD treatment compared to TID treatment (Table 2). Total cortisol exposure, the area under the serum concentration-time curve over 24 hours (AUC_0-24h_) and partial exposure estimates (AUC_0-4h_, AUC_4-10h_, AUC_10-24h_) are also displayed in Table 2. Total cortisol exposure (AUC_0-24h_) was lower during OD than during TID treatment. During the first 4 hours after oral administration (AUC_0-4h_), cortisol exposure was similar with both OD and TID treatment but, thereafter, reduced during OD compared with TID treatment. Total exposure (AUC_0-24h_) and partial exposure (AUC_10-24h_) were approximately 18% lower with OD than during TID treatment.  Autocorrelation (AUTO) values, assessing variability of the serum cortisol time profiles, ranged from 0.7 to 0.95 during both treatments (Fig. 1, Fig. 2). Higher AUTO values indicate lower variability. The serum cortisol profiles showed significantly lower variability during OD treatment, with AUTO values closer to 1, compared to TID treatment (*P* = 2.2 × 10^–16^) (Fig. 2).

**Table 2:** Clinical characteristics after 12 weeks of treatment and serum cortisol AUC values from 24-hour sampling day.

Parameter	Treatment regimen		Difference between TID and OD	*P* value
	OD (n =16)	TID (n = 18)		
BMI, kg/m^2^	26.2 (3.5)	26.3 (3.3)	–0.353 (0.622)	.058
Systolic BP, mmHg	123 (17)	128 (17)	–5.31 (10.06)	.049
Diastolic BP, mmHg	73 (12)	77 (10)	–3.44 (6.01)	.049
HbA_1c_				
%	4.88 (1.0)	5.02 (1.3)	–0.181 (0.560)	.045
mmol/mol	29.8 (12.5)	31.4 (9.8)	1.6*	
Cortisol AUC_0-24h_, nmol/L h	3851 (845)	4698 (965)	–970 (1118)	.0084
Cortisol AUC_0-4h_, nmol/L h	2100 (318)	1996 (316)	94.1 (182.4)	.095
Cortisol AUC_4-10h_, nmol/L h	1283 (608)	1782 (488)	–561 (690)	.012
Cortisol AUC_10-24h_, nmol/L h	468 (439)	921 (587)	–503 (603)	.0084

Results are shown as mean (SD).

^*^The mmol/mol difference is calculated from the mean HbA_1c_ values.

Abbreviations: AUC, area under the serum concentration-time curve; AUC_0-24h_, AUC from zero to 24 hours; AUC_0-4h_, AUC from zero to 4 hours; AUC_4-10h_, AUC from 4 to 10 hours; AUC_10-24h_, AUC from 10 to 24 hours; BMI, body mass index; BP, blood pressure; HbA_1c_, glycated hemoglobin; OD, once daily; SD, standard deviation; TID, 3 times daily.

### Metabolomics

Through gas and liquid chromatography-mass spectrometry (GC-MS and LC-MS) analysis of serum and urine, 2406 metabolites and metabolite features were detected. From GC-MS and targeted LC-MS analyses, 478 metabolites were detected and 450 of them (94%) were putatively annotated. A total of 773 metabolites were associated with total cortisol exposure and 477 metabolites were associated with the cortisol time profile variability. Among them, 205 metabolites were overlapping, indicating association with both total exposure and time profile variability (Table 3).

**Table 3:** Total number of metabolites detected from GC-MS and LC-MS analyses of serum and urine.

Analytical method	Sample	No. of metabolites detected	No. of metabolites correlated with total cortisol exposure (AUC_0-24h_)	No. of metabolites correlated with the cortisol time profile variability (AUTO)	No. of overlapping metabolites
GC-MS	Serum	126	55	24	12
LC-MS targeted	Serum	209	87	49	21
LC-MS untargeted	Serum	1938	610	402	172
LC-MS targeted	Urine	143	21	2	0
Total	–	2406	773	477	205

Abbreviations: AUC_0-24h_, AUC from zero to 24 hours; AUTO, autocorrelation; GC-MS, gas chromatography-mass spectrometry; LC-MS, liquid chromatography-mass spectrometry.

### Serum GC-MS

Two hundred and sixty-two serum samples from 17 patients were analyzed successfully with GC-MS. One hundred and twenty-six metabolites were detected and, after standardized quality control, 119 unique metabolites were identified and underwent further analyses. Fifty-five metabolites were correlated with total cortisol exposure and 24 with the cortisol time profile variability. Among them, 12 metabolites were overlapping and correlated with both total exposure and time profile variability (Table 3).

### Serum LC-MS

Two hundred and seventy-three serum samples from 17 patients were analyzed successfully with LC-MS. Targeted analysis detected 209 metabolites and, after standardized quality control, 201 unique metabolites were identified and underwent further analyses. Eighty-seven metabolites were correlated with total cortisol exposure and 49 with the cortisol time profile variability: 21 of them were overlapping and thus correlated with both total exposure and time profile variability of serum cortisol (Table 3). The untargeted approach identified 1938 metabolite features: 610 of them were correlated with total cortisol exposure and 402 with the cortisol time profile variability. These were not identified beyond mass and peak retention time at this time point and did not undergo further analyses.

### Urinary LC-MS

Thirty-five 24-hour urinary samples from 18 patients were analyzed successfully with targeted LC-MS. One hundred and forty-three metabolites were detected and, after standardized quality control, 140 unique metabolites were identified and underwent further analyses. Twenty-one metabolites correlated with total cortisol exposure and two with the cortisol time profile variability. No metabolites were overlapping (Table 3).

### Metabolites and pathways uniquely correlated to total cortisol exposure

**Figure 3:**
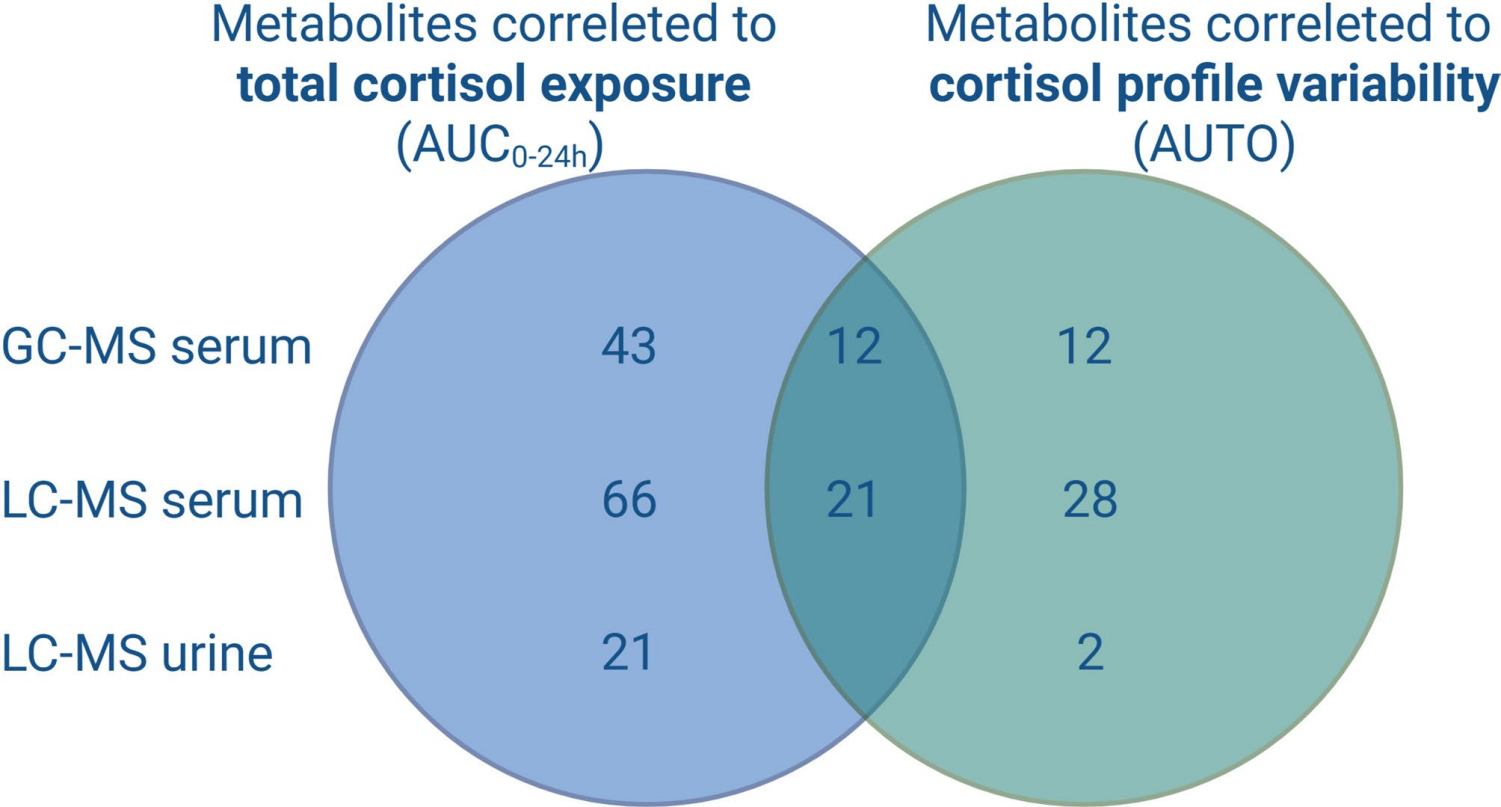
Venn diagram showing the number of detected metabolites uniquely correlated with total cortisol exposure and the cortisol time profile variability, respectively. Metabolites in both serum and urine are presented from both GC-MS and targeted LC-MS analyses. A total of 109 metabolites in serum and 21 in urine were uniquely correlated with total cortisol exposure. A total of 40 metabolites in serum and two in urine were uniquely correlated with the cortisol time profile variability. Abbreviations: AUC_0-24h_, area under the concentration-time curve from zero to 24 hours; AUTO, autocorrelation; GC-MS, gas chromatography-mass spectrometry; LC-MS, liquid chromatography-mass spectrometry.

**Figure 4:**
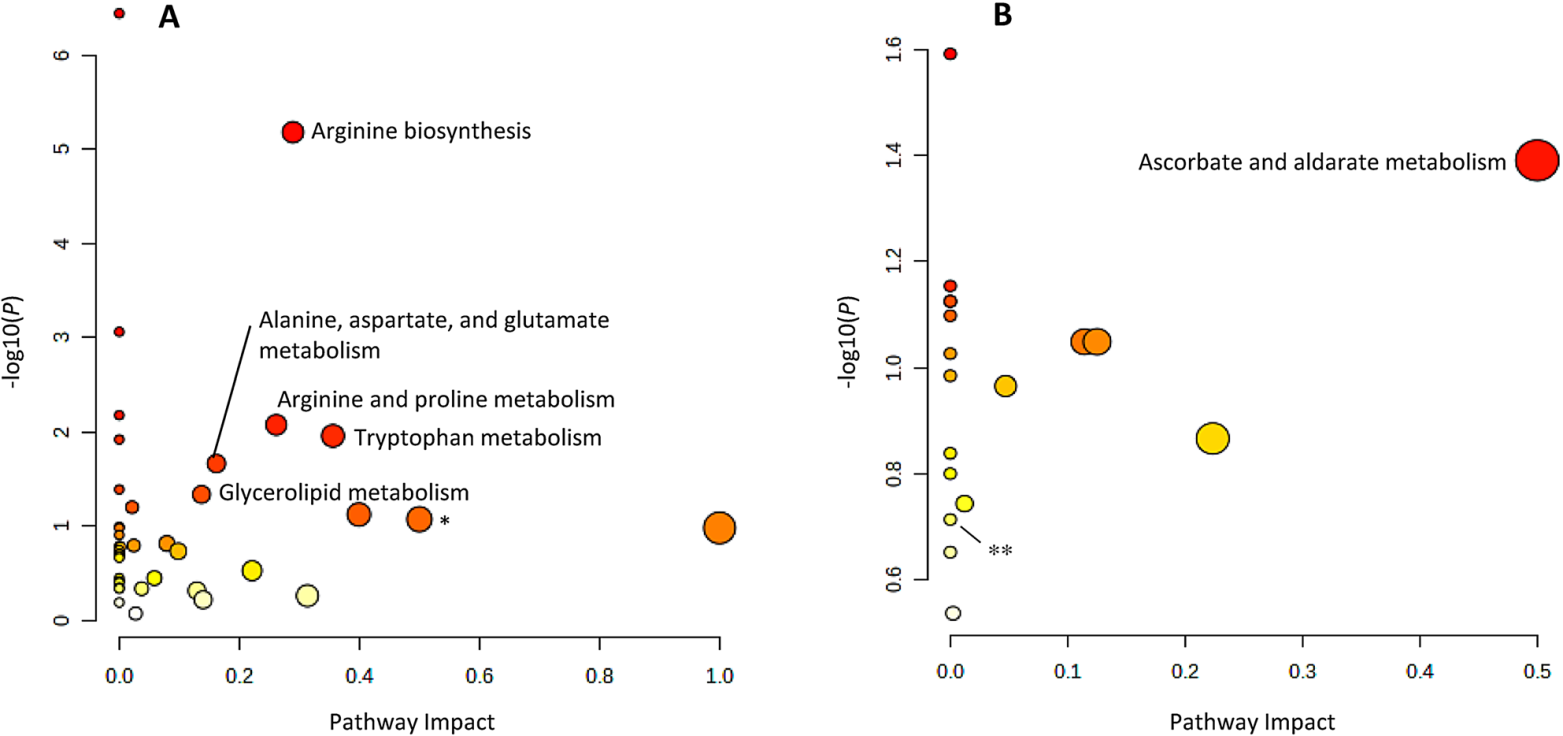
Pathway analysis of metabolites uniquely correlated with total cortisol exposure (AUC_0-24h_). The analysis shows all matched pathways, based on *P* values from enrichment analysis (y-axis) and impact values from topology analysis (x-axis). Pathways considered significant (*P* < .05, impact factor > 0) are titled in the figure. (A) shows the results for the 109 metabolites in serum that were uniquely correlated with total cortisol exposure. Five pathways reached significance: Arginine biosynthesis (*P* = 6.5 × 10^–6^, *q* = 2.7 × 10^–4^) arginine-proline metabolism (*P* = .008, *q* = .14), tryptophan metabolism (*P* = .011, *q* = .14), alanine-aspartate-glutamate metabolism (*P* = .021, *q* = .23), and glycerolipid metabolism (*P* = .046, *q* = .39). (B) shows the results for the 21 urinary metabolites uniquely correlated with total cortisol exposure. Ascorbate-aldarate metabolism turned out significant (*P* = .04, *q* = .83). *Phenylalanine, tyrosine, and tryptophan biosynthesis (*P* =.08) and **tryptophan metabolism (*P* = .19) are non-significant pathways involving the amino acid tryptophan. Metabolic pathways with higher statistical significance are plotted higher up in the graph due to the negative logarithm of *P* values on the y-axis. Darker circle colors indicate a more significant effect according to *P* value and the size of the circles corresponds to the pathway impact which is correlated to the centrality of the involved metabolites. Abbreviation: AUC_0-24h_, area under the concentration-time curve from zero to 24 hours.

In serum, GC-MS and LC-MS analyses identified 109 metabolites (43 and 66, respectively) that were uniquely correlated with total cortisol exposure (Fig. 3). Pathway analysis of these metabolites showed significance and impact on 5 metabolic pathways: arginine biosynthesis (*P* = 6.5 × 10^–6^, *q* = 2.7 × 10^–4^) arginine-proline metabolism (*P* = .008, *q* = .14), tryptophan metabolism (*P* = .011, *q* = .14), alanine-aspartate-glutamate metabolism (*P* = .021, *q* = .23), and glycerolipid metabolism (*P* = .046, *q* = .39) (Fig. 4A).

In the 24-hour urinary collection samples analyzed with targeted LC-MS, 21 metabolites were found uniquely correlated to total cortisol exposure (Fig. 3). Pathway analysis of these metabolites showed significance and impact on ascorbate-aldarate metabolism (*P* = .04, *q* = .83) (Fig. 4B).

### Metabolites and pathways uniquely correlated with variability of the cortisol time profile

**Figure 5:**
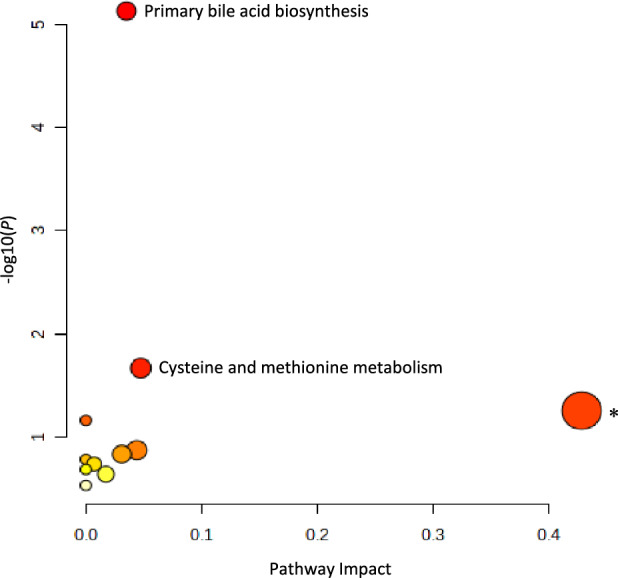
Pathway analysis of the 40 metabolites detected in serum that uniquely correlated with the cortisol time profile variability (AUTO). The analysis shows all matched pathways based on *P* values from enrichment analysis (y-axis) and impact values from topology analysis (x-axis). Pathways considered significant (*P* < .05, impact factor > 0) are titled in the figure. Two pathways reached significance: primary bile acid biosynthesis (*P* = 7.5 × 10^–6^, *q* = 6.3 × 10^–4^) and cysteine-methionine metabolism (*P* = .02, *q* = .90). *Taurine and hypotaurine metabolism (*P* = .06) is a non-significant pathway. Metabolic pathways with higher statistical significance are plotted higher up in the graph due to the negative logarithm of *P* values on the y-axis. Darker circle colors indicate a more significant effect according to *P* value and the size of the circles corresponds to the pathway impact which is correlated to the centrality of the involved metabolites. Abbreviation: AUTO, autocorrelation.

In serum, GC-MS and LC-MS analyses identified 40 metabolites (12 and 28, respectively) that were uniquely correlated with the cortisol time profile variability (Fig. 3). Pathway analysis of these metabolites showed significance and impact on primary bile acid biosynthesis (*P* = 7.5 × 10^–6^, *q* = 6.3 × 10^–4^) and cysteine-methionine metabolism (*P* = .021, *q* = .90) (Fig. 5).

Targeted LC-MS analysis of 24-hour urinary collections identified two metabolites uniquely correlated with the cortisol time profile variability: oleamide and succinic acid. No pathway analysis was performed as three or more metabolites are needed for this analysis.

## Discussion

In this study, we identified serum and urine metabolites, as well as metabolic pathways, that correlate independently with either total cortisol exposure or variability in the serum cortisol time profile. To our knowledge, this has not been demonstrated previously. Our approach was to study patients with AI treated with two different HC replacement regimens (OD vs TID), which produced differences in both total exposure and time profiles of serum cortisol (Fig. 1). Moreover, we applied a novel approach to quantify intra-day fluctuations in the cortisol profile by calculating the lag–1 autocorrelation from 24-hour serum cortisol concentrations (AUTO). This enabled us to distinguish between two key measures of cortisol dynamics; total exposure and time-profile variability.

According to the Swedish Addison Registry, the mean hydrocortisone replacement dose was 28.1 ± 8.5 mg/day, as reported in a study published in 2017^22^. Due to the observed adverse outcomes in this patient population, a replacement dose of 15–25 mg/day has been recommended^23^. The study population in the present study had a mean replacement dose of 32 mg/day (Table 1) and is therefore considered to be in line with real-world practice in Sweden at the time of the study.

The study was performed using a crossover design with identical daily doses of hydrocortisone administered either as once-daily dual-release (OD) or thrice-daily immediate-release (TID) formulations. Compared with OD treatment, the TID regimen resulted in higher total exposure and greater intra-day variability of serum cortisol concentrations. Both AUC and AUTO also showed a considerable within-group variation. Our analysis therefore focused on correlating metabolite levels with the two distinct aspects of cortisol dynamics; total exposure (AUC_0-24h_) and profile variability (AUTO) for both treatments.

We identified 109 serum metabolites specifically correlated with total cortisol exposure, with arginine showing the strongest association. Both arginine biosynthesis and arginine-proline metabolism were among the top significantly enriched pathways, with arginine biosynthesis remaining the only pathway statistically significant after FDR correction (Fig. 4A). Arginine modifies protein synthesis, alters glucose metabolism, and is a precursor of the production of nitric oxide, an important regulator of vascular tone and blood pressure^24,25^. In line with previous studies, our results show that the amino acid tryptophan and its metabolism were also correlated with total cortisol exposure (Fig. 4A). Tryptophan is metabolized by either the serotonin or the kynurenine pathway. Serotonin is an important neurotransmitter in both the gastrointestinal tract and the central nervous system, and kynurenine is a key regulator of the immune response and energy metabolism^24,26^. More recently, tryptophan has also emerged as an interesting mediator in cardiovascular biology^27^. The amino acids alanine, aspartate, and glutamate were also correlated with total cortisol exposure, and they are linked to glucose metabolism and the tricarboxylic acid (TCA) cycle, which has a central role in energy metabolism^24^. Glycerolipids are composed of mono-, di-, and tri-fatty acid-substituted glycerols^28^, and in line with previous metabolomic findings, we found this group of metabolites correlated with total cortisol exposure (Fig. 4A). The urinary metabolites, uniquely correlated to total cortisol exposure, were significantly involved in ascorbate-aldarate metabolism (Fig. 4B). Ascorbate, or vitamin C, is an important coenzyme in several metabolic pathways including noradrenaline biosynthesis and tyrosine metabolism^24^. It is also an important antioxidant and necessary to maintain connective tissue and bone health^24^. In summary, all significant metabolic pathways that we found uniquely correlated with total cortisol exposure in this study, are mainly related to various aspects of energy metabolism.

Furthermore, we found 40 metabolites in serum uniquely correlated with the cortisol time profile variability. These metabolites were mapped to two pathways in particular: primary bile acid biosynthesis and cysteine-methionine metabolism, with primary bile acid biosynthesis being the only pathway that remained statistically significant after FDR correction (Fig. 5). Primary bile acids play a key role in both lipid and glucose metabolism^29^, and research implicates important interactions between bile acids and glucocorticoid metabolism^30^. Possible links include their common precursor, cholesterol, as well as the bile acid receptors; farnesoid X receptor and the G protein-coupled bile acid receptor, both of which are mainly expressed in the liver but also in the adrenal cortex^30^. In addition, both primary bile acid and glucocorticoid secretion profiles have a physiological diurnal regulation in humans^31^. We have previously shown that an improved serum cortisol time profile induces steroid 5β-reductase activity^17^, an enzyme involved in both cortisol metabolism and bile acid synthesis^32^. Cysteine is an amino acid with antioxidant properties and is important in energy metabolism. It is also a structural component of many tissues and hormones in humans^24^. Methionine is the metabolic precursor of cysteine and several studies have shown that chronically high levels of methionine are associated with atherosclerosis and cardiovascular disease^24^.

Further, two metabolites were identified in urine that were uniquely correlated with the cortisol time profile variability: oleamide and succinic acid. Oleamide is an endogenous fatty acid amide initially considered to be a sleep-inducing molecule, and later recognized for a wide range of effects on the central nervous system. More recent data suggests that oleamide is also involved in inflammatory response in humans^24^. Succinic acid is a dicarboxylic acid and an intermediate in the TCA cycle. Recent studies have shown that it has an essential role in regulating inflammatory response^24^. The taurine-hypotaurine pathway also tended to be correlated with the cortisol time profile variability (Fig. 5). Taurine plays a key role in energy metabolism, particularly glucose and lipid metabolism, and recent studies suggest that taurine is associated with various aspects of the cardiovascular system, including blood pressure regulation, and anti-inflammatory effects^33^. Taurine is synthesized in the liver mainly via the cysteine-sulfinic pathway and increases bilirubin and cholesterol excretion in bile as it conjugates with bile acids to form bile salts^33^. In summary, the majority of the metabolic pathways uniquely correlated with the cortisol time profile variability were associated with bile acid metabolism, the inflammatory response, and cardiovascular regulation.

The study is a post-hoc analysis of a subgroup of participants from a previous study and, as such, it has limitations. Serum sample collection for metabolomic analysis was carried out at the start of each treatment period and therefore reflects only the short-term effects of change in total cortisol exposure and the cortisol time profile variability, which could result in a carry-over effect not accounted for in our analysis. Furthermore, given the recently highlighted limitations regarding metabolomics-based pathway analysis^34^, our results from pathway analysis should be interpreted as hypothesis-generating. A strength of the study is the 24h in-house setting and use of standardized meals for all participants during both study periods, which minimized the impact of food intake on the metabolomic outcome. We also restricted the pathway analyses to metabolites that were both confidently identified and assigned a unique Human Metabolome Database identifier (HMDB ID), to minimize over-interpretation.

## Conclusion

Our study identified different groups of circulating and urinary metabolites uniquely correlated with either total exposure or variability in the time profile of serum cortisol. These metabolites are involved in a diversity of metabolic pathways related to energy metabolism, primary bile acid synthesis, inflammation, and cardiovascular function, reflecting the broad impact of glucocorticoid action. Our findings support that total exposure and the intra-day variability of the serum cortisol profile likely exert different metabolic- and regulatory effects in the human body. The outcome of this study may contribute to the important challenge of identifying novel biomarkers of glucocorticoid action and could potentially guide development of more individualized hydrocortisone therapy.

## Methods

### Study design

This is a hypothesis-generating study based on a previously published open, randomized, controlled, 12-week, crossover, clinical trial comparing an OD dual-release HC tablet to the same daily dose administered as conventional HC tablets 3 times daily (TID) in patients with primary AI^15^. The study protocol (EudraCT: 2006-0007084-83; www.ClinicalTrials.gov ID: NCT00915343, first posted on 08/06/2009) was approved by the Ethics Committee in Gothenburg (Dnr: 104-07 and T295-16), and by the Swedish Medical Product Agency. The study was performed according to the principles of Good Clinical Practice and the Declaration of Helsinki. Informed patient consent was obtained before entering any part of the study.

### Study participants

This study included a subpopulation of 18 patients who all underwent full 24-hour in-house pharmacokinetic sampling^15^. Briefly, patients diagnosed with primary AI > 6 months prior to study entry were included. All patients had received stable GC replacement therapy with HC 20-40 mg/day for ≥ 3 months prior to study entry and the dose was kept constant throughout the study. Patients on a twice-daily conventional HC therapy regimen were converted to a TID regimen 4 weeks before study entry with the same total daily dose. Any medications or agents that might interfere with cortisol distribution (metabolism and transport) were discontinued within 14 days prior to study start. Patients with ongoing treatment with dehydroepiandrosterone and oral estrogens were excluded. Mineralocorticoid and/or levothyroxine replacement therapy had to be stable for ≥ 3 months before inclusion and during the entire study.

### Study protocol

The protocol was previously described in detail by Johannsson G et al^15^. Briefly, patients were randomized into two 12-week treatment periods to receive dual-release HC treatment OD and conventional HC treatment TID in a crossover manner. All patients underwent a standardized in-house pharmacokinetic sampling for serum cortisol profiling over 24 hours at the beginning of each treatment period. Blood samples were collected over the 24-hour period and 24-hour urine samples were collected on these sampling days as well. Serum samples from eight time points (baseline and 25min, 2h, 4h, 8h, 12h, 16h and 24h post first tablet intake) were used for cortisol and metabolite analysis in this study. Patients remained in the clinical trial unit during the entire sampling day and received standardized meals at pre-defined time points. OD dual-release HC tablets were administered orally in a fasting state in the morning at 0800 h and the TID conventional HC tablets were administered orally at 0800, 1200, and 1600 h. Body weight, height, glycated hemoglobin (HbA_1c_), and blood pressure were measured at baseline and at the end of each 12-week period. Serum and urine samples were stored at –80°C until the analysis of metabolomics. All analyses were performed in one run.

### Laboratory methods

Serum cortisol was assayed by a competitive immunoassay using direct chemiluminescent technology (ADVIA Centaur; Bayer Diagnostics, Femwald, Germany). The sensitivity of the assay was 5.5 nmol/L and the total coefficient of variation was < 8%.

Metabolites were detected in serum using gas chromatography-mass spectrometry (GC-MS) and liquid chromatography-mass spectrometry (LC-MS), and in urine using LC-MS. Samples belonging to the same study subject were run in close order to reduce run-order issues. All metabolomic analyses were carried out at the Swedish Metabolomics Centre in Umeå as previously described^19^. Metabolites were detected based on accurate mass and retention time relative to calibration metabolite standards for LC-MS and retention index for GC-MS. Peaks with low response and/or bad chromatography were excluded. Metabolite features detected with GC-MS were identified by matching with a pre-defined list (in-house and Max Planck libraries, Swedish Metabolomics Centre). LC-MS analysis was performed in both positive and negative ionization modes, and in a targeted and untargeted approach. With targeted processing, metabolites were putatively identified by searching within the data for specific metabolites from a pre-defined list (in-house libraries, Swedish Metabolomics Centre). With untargeted processing, metabolite features were searched for in an untargeted fashion with a peak-picking approach. The detected untargeted features were annotated as “measured neutral exact mass@retention time on column” and were not identified further. All detected metabolites were included in association analyses. For the final pathway analyses, only metabolites confidently identified Level 1, according to metabolite identification standards^35,^ and assigned a unique Human Metabolome Database identifier (HMDB ID) were included.

### Pre-processing of metabolomic data

Metabolomic data was pre-processed as compound concentration data using MetaboAnalystR^36,37^. Annotated compounds with missing or zero values were replaced per standard MetaboAnalystR processing. Metabolites were filtered based on relative standard deviation (SD), removing uninformative features that have low variation between samples. Samples were quantile normalized and log-transformed, and metabolites were auto-scaled in line with established methods previously described^38^.

### Pathway analysis

The web-based tool for analysis, integration and visualization of human metabolomic data, MetaboAnalyst5.0, was used for pathway analysis of the metabolites across the different groups^36^. Briefly, pathway analyses is conducted using two complementary approaches: pathway enrichment analysis and pathway topological analysis. Enrichment analysis applies over-representation analysis to determine whether differentially expressed metabolites (DEMs) are associated with particular pathways more frequently than expected. Topological analysis evaluates the centrality and betweenness of metabolites in a given metabolic network (directed graph) to estimate their importance in the organization of the network. A pathway impact score is then derived from this analysis as the sum of the importance measures of DEMs, normalized by the sum of the importance measures of all metabolites within each pathway. Pathway annotation was performed using the *Homo sapiens KEGG* pathway library (Kyoto Encyclopedia of Genes and Genomes)^39–41^. Only metabolites assigned a unique Human Metabolome Database identifier (HMDB ID), were included in the pathway analysis. Pathways with a raw *P* value <.05 from pathway enrichment analysis is presented as significant. To account for multiple testing false discovery rate (FDR)–adjusted *P* values (*q* value) are reported for significant pathways as well. The significance threshold for FDR correction was set at .05.

### Analytical approach

We developed a novel approach to investigate the metabolome associated with either total cortisol exposure and/or the intra-day variability in the cortisol time profile, using two different hydrocortisone (HC) treatment regimens (Fig. 1). Total cortisol exposure and the profile variability were calculated from 24-hour serum cortisol concentrations for each participant for each treatment. Total exposure was calculated by the area under the serum cortisol concentration curve from zero to 24 hours (AUC_0-24h_), using the trapezoidal integration method via the R package pracma^42^. Variability of the cortisol time profile was determined by calculating the autocorrelation (AUTO) for the serum cortisol values using the R package stats^43^. AUTO is the correlation of a variable (serum cortisol) in a time series against a lagged version of itself (i.e. comparing values at T with T+1 across the time series). This approach quantifies the intra-day fluctuations in serum cortisol concentrations, enabling an individualized assessment of cortisol time profile variability rather than grouping patients solely based on treatment regimens. For each participant and treatment, an AUTO value was calculated as a measure of curve variability. A value near 1 indicates a more stable cortisol time profile with less fluctuation. The relationship between the total cortisol exposure (AUC_0-24h_), the cortisol time profile variability (AUTO) and metabolite expression was assessed using a Bayesian generalized linear model via the R package rstanarm^44^. The posterior distributions of each model was generated using the R package insight^45^ and describes the effect size of the linear relationship between a metabolite and the cortisol dynamics (AUC_0-24h_ and AUTO). The 89% credible interval (CI) of the posterior distributions was calculated using the highest density interval approach^46^ and significance^47^ was applied to those metabolites for which the 89% CI of the posterior distribution did not include 0. This approach allowed us to identify metabolites that were significantly correlated with changes in total exposure and/or changes in the time profile variability of serum cortisol.

### Statistical analysis

As previously^15^, for analyses of cortisol pharmacokinetics as well as biochemical and clinical variables, within-patient differences between OD and TID treatment were calculated. To assess potential sequence effects, these differences were then compared between patients who started on OD versus those who started on TID using Fisher’s non-parametric two-sample permutation test or Wilcoxon signed-rank test for continuous variables, and the sign test for ordinal and dichotomous variables. This approach allows evaluation of whether the treatment sequence influenced the observed differences. All significance tests were two sided and conducted at a significance level of .05.

## Data Availability

The metabolomics dataset generated in this study has been deposited to the MetaboLights^48^ repository with the study identifier MTBLS13147 at [https://www.ebi.ac.uk/metabolights/MTBLS13147] . All data supporting the findings of this study are available from this repository.
